# Hospital and Health News

**Published:** 1923-11

**Authors:** 


### HOSPITAL AND HEALTH NEWS.

### India's "Baby Week."

Lady Reading's "National Baby Week" to be held in India in January next has received a great deal of public support, especially from the Indian States.

### A "Robert Burns" Cot.

A cot has been endowed, or with its aid will soon be endowed, at the Children's Hospital, Birmingham, by the Burns Club of the city, which has sent £840 toward a "Robert Burns" Cot.

### Work for Dentists.

Sir Kingsley Wood, in opening the Dental Exhibition, remarked that there are now in England and Wales 3,000,000 children in need of dental treatment, 500,000 of them urgently.

### Old X-Ray Plates.

It will be of considerable interest to hospitals to know that good prices can be obtained for old X-ray plates. Any institution which has plates to dispose of should communicate with Messrs. Bowen Bros., of 186-188, Liverpool Road, Islington, N. 1.

### Appointments at the Ministry of Health.

Sir William Joynson-Hicks has appointed Lord Erskine, M.P., to be his Parliamentary Private Secretary, Mr. Douglas Veale, of the Ministry of Health, to be his Private Secretary, and Mr. A. Nevil Rucker, of the Ministry of Health, to be his Assistant Private Secretary.

### Contribution Scheme for the Potteries.

After a Conference, addressed by Mr. S. R. Lamb, Secretary of the Sheffield Hospitals' Council, the National Council of the Pottery Industry has appointed a committee to form a provisional contribution scheme for the Potteries on lines similar to those proving successful at Sheffield.

### Hospital Light Verse.

A set of light verses, showing remarkable metrical skill, appeared in the August number of *St. Thomas's Hospital Gazette*, under the title "To Harold," and over the initials R. W. S. Knowing how heavy light verses usually are, the reader familiar with the "Oxford Book of English Light Verse" will turn to this poem with pleasure.

### The Chadwick Lectures.

The Chadwick Public Lectures are being delivered during October, November and December, and the lecturers include the Medical Officers of Health at Leicester, Glasgow, and the Medical Officer of Health at the Port of London. All particulars as to time and place can be obtained from the Secretary at the Offices of the Chadwick Trust, 13 Great George Street, S.W. 1.

### Mr. E. W. Morris as Hospital Historian.

We hope that none of our readers has missed the delightful articles that Mr. E. W. Morris, C.B.E., Secretary of the London Hospital, has been contributing to the *Daily Telegraph*. They are not appeals. They began with historical sketches of the oldest London foundations, written in the light and lucid style which Mr. Morris made his own in his short "History of the London." The *Daily Telegraph* is to be congratulated on the series and on the brilliant contributor whose pen it has secured.

### Awards for the Highbury Fire.

Messrs. James O. Ayers, W. T. Jackson, Percy G. Sharp, Alfred P. Thorpe and Robert W. Bradley, all of Birmingham, have been awarded Bronze Medals and five guineas each for saving or trying to save life in the serious fire that occurred in the open-air ward of the Ministry of Pensions Hospital, Highbury, on June 13, when two disabled patients lost their lives. The awards are made by the Society for the Protection of Life from Fire.

### The Late Sir William Treloar.

Suggestions having been made for a memorial to the late Sir William Treloar, and the matter having been considered by the Trustees of the Lord Mayor Treloar Cripples' Hospital and College, at a meeting under the chairmanship of Lord Burnham, it was decided that the present time is inopportune for the raising of a memorial fund. It is hoped that the public will look upon the Hospital and College as a great Trust bequeathed to them by the late Sir William Treloar, and give such support that the Institution may be carried on as a permanent memorial. The best help would be by annual contributions.

### A Butterless Hospital.

Mr. H. W. Burleigh, Secretary of the Hospital for Epilepsy and Paralysis, Maida Vale, has taken the unusual step of transferring a "controversy raging at this hospital" from the privacy of its earless walls to the columns of the weekly newspapers. The controversy concerns "Butter or Margarine," which last is all the hospital can afford either patients or staff at present. The moral for the general public is obvious, and we congratulate Mr. Burleigh on his ingenious presentation of it.

### Self-Controlled Air Cushions.

When Papyrus went to America he was sent in a specially constructed loose box built for the occasion by the Self-Controlled Air Cushion Company, Ltd., of Sentinel House, Southampton Row, lined throughout with specially made self-controlled air cushions of a thickness of about 10 inches all round the compartment. These cushions are made on the same principle as the patent Self-Controlled Air Bed which, during the last year, has been adopted by most hospitals and public institutions throughout the country for the class of patient usually placed on a water-bed. Motor-cars are also being upholstered with self-controlled air cushions filled with pure air.

### A Warwickshire Sanatorium.

The new Sanatorium for Tuberculosis at Hertford Hill, Warwick, erected at a cost of £70,000 as a memorial to King Edward VII., was dedicated recently by the Bishop of Coventry. Splendidly situated and admirably planned, the Sanatorium will, when finished, be one of the finest buildings of its kind in the country. The formal opening will probably take place some time next year. Grants towards the cost of the Sanatorium are made by the Government and the Red Cross Society, whilst Alderman J. A. Watson, of Chadwick Manor, earned the gratitude of the county when he offered to defray the cost of blocks at a figure between £25,000 and £27,000.

### Longton Cottage Hospital.

Sir Francis Joseph, C.B.E., has opened the new out-patient department of Longton Cottage Hospital, which, by the way, will relieve the North Staffordshire Infirmary. This is the first step in the extensive additions that have been planned. Mr. J. H. Beckett, of Longton, is the architect. A sum of £5,000 is the cost of this extension, and £12,000 more will be needed to complete the rest of the scheme. But the hospital has kept out of debt and not made a special appeal for some years. Mr. Harry Aynsley, J.P., the son of the late Mr. John Aynsley, the hospital's founder, is the president.

### Lectures on Food and Diet.

A series of lectures upon "Food and Diet" will be delivered at the Institute of Hygiene on Tuesday, November 6, and the three following Tuesdays. The subjects are:—"Preservatives and Adulterants in Foods," by James Fenton, M.D., D.P.H.; "Pure Food Supplies," by A. Middleton Hewat, M.D., D.P.H.; "Diet and Disease," by Sir Kenneth Goadby, K.B.E., M.R.C.S., D.P.H., L.D.S.; "Habits, Diet and Malignant Disease," by J. E. Adams, M.S., F.R.C.S. Each lecture will begin at 3.30 p.m. and seats can be reserved on application to the Institute at 33-34 Devonshire Street, W.1.

### Sunderland Royal Infirmary.

The new Operating Theatre Block at Sunderland Royal Infirmary, built and equipped by his family in memory of the late Mr. J. Y. Short, J.P., ship owner, at a cost of £7,000, was opened recently by his widow and his son, Mr. T. Short. Dr. Wm. Robinson, Chairman of the Medical Board, described the new building, and said that the site had been carefully selected so that the block would not interfere with the future extension of the wards of the infirmary, which was much needed because of the great length of the waiting list. The block consists of a suite of eleven rooms opening off a central corridor, and includes a dark room for examination of the eye, ear, throat and nose, cystoscopy, etc., and in which mastoid and similar operations can be performed; surgeons' preparation room with six lockers, a long and a shower bath; an anæsthetising room; two large theatres, in which part of the roof and the whole of one side is of glass. Dr. Robinson showed that these two additional theatres would admit of a throat and ear department being established, and also of the increase in size of the eight old wards which was necessary in the future.

### Keighley Victoria Hospital.

In the middle of October the Duchess of Devonshire opened a bazaar in aid of the Keighley Victoria Hospital. In connection with it Mr. W. A. Brigg has written a "Life Story" of the hospital. The handbook covers the period from the opening of the cottage hospital in a house at Highfield in 1874, when the kitchen table became the operating table of the institution. With eight beds at her disposal Mrs. Stead, the matron, was in charge for seventeen years. In 1892 the Mansion House in Victoria Park was proposed, says Mr. Brigg, for a general infirmary, to which the cottage hospital, had this plan been adopted, would have served as a convalescent home. Instead Highfield was extended by thirty-two beds, and in the Diamond Jubilee year the hospital was rechristened. A new wing was added in 1904. Miss Garner, the present matron, was appointed in 1912.

### A Hospital Scheme for Croydon.

The Board of Management of Croydon Hospital has issued an appeal for £250,000 to erect and equip an up-to-date hospital. The present hospital is hindered by cramped and inadequate premises. In order to bring the appeal prominently before the people of Croydon and district, in which there are 950 roads, a placard will be placed in each thoroughfare asking the residents in that particular road—which will be named on the notice—to contribute towards the cost. It is expected that this will lead to a competition between the various streets and roads, with the result that an adequate sum will be raised. The idea of the Board is to make provision for two hundred beds and to establish a fully-equipped out-patients' department.

### Hospital Saturday Fund.

The collections in the Metropolis on behalf of the Hospital Saturday Fund on "Hospital Saturday," October 6, were, we understand, of a satisfactory character, but figures are not at present available. As in former years there was no street collection, but hearty co-operation was given by the railway companies, theatres, cinemas, football clubs and business houses. Thirty Local Committees of the Fund worked zealously and obtained good results. It is interesting to note that, notwithstanding industrial depression, the workshop and business house collections are slightly in excess of last year, which clearly shows the confidence reposed in the Fund. The work of the Fund can be to some extent realized by the fact that over 1,300 subscribers are assisted weekly with Hospital, Surgical, Dental or Convalescent Home treatment.

### An American Hospital in London.

*The Hospital World*, of Toronto, states that a permanent committee has been formed, consisting of representatives of all American and Anglo-American Societies, to found an American Hospital in London, staffed by American doctors and nurses, to minister to the needs of the 12,000 American residents in Great Britain and to the perennial stream of American visitors from the States. The success of the American Hospital in Paris has begotten this desire to repeat the experiment in London. Consul-General Skinner is chairman of the Committee. Patients will contribute or not, according to their means. There is expected to be little difficulty in finding the necessary money.

### "Yadil" Antiseptic.

An antiseptic which is becoming well thought of by medical men is "Yadil," a preparation of purely vegetable origin and entirely harmless, its active principle being natural essential oil of garlic. It is claimed that "Yadil" disinfects the whole human system, internally and externally, without the slightest risk of injury to the most delicate internal organs. It is absorbed into the blood stream and reaches every part of the body, destroying disease-germs wherever they may be. For more than a year, from early in 1915 to the middle of 1916, "Yadil" was subjected to severe private tests to ascertain its effectiveness, its safety, and its stability. For over five years thereafter, from November, 1916, to the beginning of 1922, it was kept exclusively for the medical profession. During that period, thousands of doctors used it personally, gave it to their families, and prescribed it for their patients. Those who would like to hear more of this preparation, and of its widely varied uses, should write Messrs. Clement and Johnson, Ltd., of 19 Sicilian Avenue, W.C. 1, who publish an attractive little "Yadil Book" and much other matter on the subject.

### London Medical Exhibition.

The London Medical Exhibition was held at the Central Hall, Westminster, from October 1 to 5. The exhibits included hospital furniture, surgical instruments and various miscellaneous appliances. A home set of violet rays was shown on precisely the same lines as those installed in hospitals and consuming less current than an ordinary lamp. The process of producing insulin was one of the most interesting demonstrations. There are eleven stages in the process, and these are illustrated from the ox pancreas to the finished insulin ready for issue. The demand for insulin is ever-increasing, and as that occurs the cost happily decreases. A blood taximeter for recording blood pressure attracted particular attention, and the Exhibition was deservedly well attended by medical men from London and the provinces, and also from the Dominions overseas.

### Strowger Automatic Telephones.

The Strowger Automatic Telephone Service, which is a thoroughly up-to-date and efficient system, has been installed in the new London County Hall. The new installation will afford speedy, accurate and private inter-communication between some 650 different departments in the building, and also gives access, through an associated manual telephone switchboard, to the public telephone exchange system and the many outlying districts and local Borough Administrations which come under the jurisdiction of the London County Council. The Exchange operates on what is known as a three digit basis. The act of calling any number on the system entails three successive movements of the dial attached to the telephone in use by the calling party. This manipulation by the finger tip is, however, extremely simple and occupies six seconds only. Assuming the person called to be disengaged, the response is immediate, and in the event of an engaged line a distinctive audible signal is at once heard in the receiver at the calling station. Clearing at the conclusion of a conversation follows the replacement of the receiver upon the switch-hook and is instantaneous, the line therefore being immediately available for another call. The entire equipment has been manufactured and installed on behalf of the British Post Office Telephone Department by Automatic Telephone Manufacturing Co., Ltd., Liverpool. This Automatic Telephone Installation constitutes the largest Exchange of its kind in the Empire.

### The Blind Masseur.

Speaking recently at the General Meeting of the Association of Certificated Blind Masseurs, Sir Robert Jones, President of the Association, said that its progress was due to the splendid energy and undaunted perseverance of members of the Association. It pointed to yet greater developments in the future. At the termination of its fourth financial year the Association numbered 160 members, of whom 81 were ex-soldiers, 37 civilians, and 42 masseuses. The general public and even some of the medical profession were badly in need of information about Massage and the Blind Masseur. There was a popular idea that the Association was of the nature of a transient war charity, but there was no evidence of this in the figures, which showed that 71 of the 160 members were civilians. Secondly, out of this misconception had arisen the belief that—however excellent a blind masseur might be, and however patriotic it might be to employ him—he could not, of course, equal the skill of a masseur with sight. That the Blind were specially suited to become masseurs was now a commonplace amongst all who had supervised their work. They acquired in certain sympathetic directions a deftness and delicacy of touch not often found in the ordinary masseur—they worked by insight and that curious compensation of nature, by which where one faculty was weakened another was made strong.

